# An Atypical Case of Lyme Disease Presenting With Lyme Carditis

**DOI:** 10.7759/cureus.35907

**Published:** 2023-03-08

**Authors:** Usman S Najam, Adnaan Sheikh

**Affiliations:** 1 Internal Medicine, University at Buffalo Jacobs School of Medicine and Biomedical Sciences, Buffalo, USA; 2 Internal Medicine, Arnot Ogden Medical Center, Elmira, USA

**Keywords:** av block, cardiovascular implications, infectious disease pathology, zoonotic infections, lyme carditis, lyme's disease

## Abstract

Lyme carditis is a rare but serious manifestation of Lyme disease presenting in the early disseminated stage of the disease often after a diagnosis has already been discovered. The classic case of Lyme disease presents a patient who had been participating in outdoor activities in a Lyme-endemic region and was found to have a tick bite. These patients often present in the early localized stage, within the first 1-2 weeks with the well-recognized erythema-migrans rash or with generalized flu-like symptoms. Here we describe a case of a 70-year-old male who presented to the hospital without any typical Lyme disease prodrome but instead with generalized symptoms of progressive orthopnea and dyspnea on exertion. His original diagnoses were not of infectious origin however after incidentally being found to have a second degree, Mobitz type 1 atrioventricular (AV) block; Lyme tests were ordered, and a diagnosis was confirmed. This incidence shows the importance of having a Lyme disease diagnosis when regionally appropriate for patients who may present with no other signs or symptoms other than an AV block. As in this case, after a diagnosis has been made the management becomes the treatment of the infection rather than the treatment of the symptoms themselves.

## Introduction

Lyme disease is a tick-borne infection mostly found in the Northeast and upper Midwest United States with 95% of confirmed cases reported from only 14 states [1]. It is transmitted by a bite from the Ixodes Scapularis tick which is host to the microaerophilic Gram-negative spirochete Borrelia Burgdorferi. Even though Lyme disease is the most common vector-borne disease in the United States, it is often thought of as an uncommon disease due to its 30,000 cases reported per year. However, it is likely much more common with an estimated occurrence of approximately 476,000 cases per year [1]. Its ability to be underreported may be due to its various presentations, often atypical with minimal or no symptoms. It varies from one patient to the next and while it may appear asymptomatic at first, in many patients its symptoms can present weeks, months, or even years later with Lyme carditis, Migratory arthralgias, Neuroborreliosis, etc. Lyme carditis is a rare manifestation that occurs in 1.5%-10% of untreated adult patients in the United States [2]. Here we report a case of an elderly male with an atypical presentation of Lyme disease who presented with various degrees of atrioventricular (AV) blocks on an electrocardiogram (EKG).

## Case presentation

We present a 70-year-old Caucasian male with a relevant past medical history of hypertension and calcific aortic stenosis who presented to our emergency department with progressive orthopnea and dyspnea on exertion. The patient had initially presented with these symptoms to his primary care physician who was concerned the patient’s bicuspid aortic valve stenosis had worsened and was therefore instructed to the hospital for further workup. The patient's last echocardiogram was done nearly two decades prior which at that time showed a left ventricular ejection fraction of 60% and moderate aortic insufficiency due to a bicuspid aortic valve. Upon initial presentation in the ED, the patient's labs were significant for an increased erythrocyte sedimentation rate of 136, white blood cell count of 16.6, hemoglobin of 9.3, creatinine of 2.6, and normal liver enzymes. Troponins were negative but his brain natriuretic peptide was elevated at 877. His admitting EKG was significant for bradycardia with a heart rate in the mid-40s and a first-degree AV block. The patient was admitted for further monitoring and workup.

On physical exam, the patient appeared very dyspneic although saturating well on ambient air. He was found to have noticeable jugular venous distension, a significant early systolic murmur at his right upper sternal border, and bilateral pedal edema to his ankles. Upon further lab workup, his procalcitonin returned elevated at 0.49 ng/mL. His chest x-ray showed mild right basilar hypo-aeration, computerized tomography (CT) chest imaging was significant for a small anterior pericardial effusion, and echocardiogram was mostly unchanged from previous studies showing a left ventricular ejection fraction of 50-55%, and moderate aortic stenosis. The patient had an exercise stress test in which 77% of the maximal predicted heart rate was achieved. He had to stop the testing due to severe dyspnea but without any chest pain. There was a hypotensive response to stress with an abnormal ST segment suggestive of cardiac ischemia which the patient at the time deferred to an outpatient workup.

**Figure 1:**
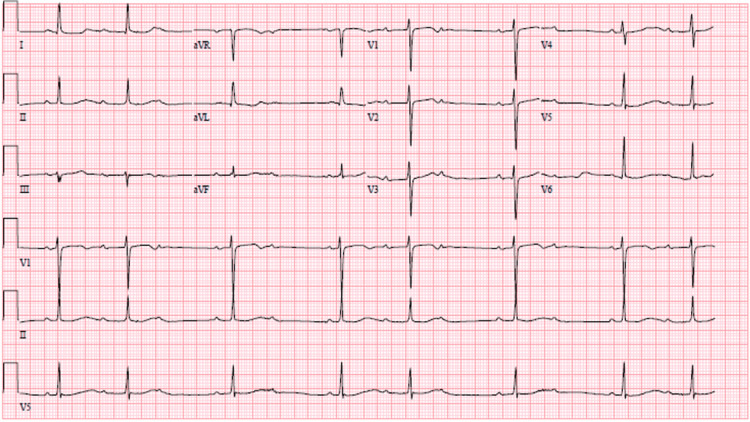
Second degree, Mobitz type 1 AV block

It was felt at the time his symptoms were multifactorial with possible etiologies including his anemia, progressive kidney disease, possible congestive heart failure exacerbation, worsening aortic stenosis, and/or pneumonia. The patient did not have available labs in the last decade for comparison and for this reason, clinical judgment was made without a reference point. Due to the prevalence of Lyme disease in the northeast and the patient’s symptomatology, a tick panel was ordered which came back positive for Lyme (Table 1) as well as Ehrlichia Chaffeensis (Table 2). On telemetry, the patient was noted to have various degrees of atrioventricular blocks, including first-degree AV block, second-degree, Mobitz type 1 AV block (Figure 1), and occasional 2:1 block. Electrophysiology was consulted and initially felt the patient would benefit from a pacemaker, however when presented with a positive Lyme titer, it was suggested to treat the underlying Lyme disease and monitor progression with a loop recorder. The patient was started on first-line treatment with intravenous Rocephin to which he responded reasonably well. Within three days of treatment, the patient's PR interval shortened from an initial 330 msec to 270 msec and was otherwise stating much improvement in his dyspnea and orthopnea. As the patient symptomatically improved during this time, he insisted on going home despite attempts to convince him otherwise by the internal medicine team, his primary care physician, and his wife. He ultimately left against our medical advice but was advised if he had symptoms of lightheadedness, dizziness, shortness of breath, chest pain, or palpitations he should return to the hospital immediately. The patient was discharged with Doxycycline for 21 days in the treatment of his Lyme disease. The patient did not return to the hospital after discharge, however followed up with an outpatient provider. On his three-week follow-up, after completion of his antibiotics, he was told by his outpatient provider he had reverted to normal sinus rhythm without AV block along with near resolution of his symptoms.

**Table 1:** Lyme antibodies

Order Comment	Ordered by Lab
Lyme WB IgG Ab	Positive
Lyme WB IgG Ref Com	Reference range: Negative
Lyme WB IgM Ab	Positive
Lyme WB IgM Ref Com	Reference rage: Negative
Band(s) present IgG	93, 66, 58, 45, 41, 39, 30, 28, 23, 18 kDa
Interpretive information IgG immunoblot	For this assay, a positive result is reported when any 5 or more of the following 10 bands are present: 18, 23, 28, 30, 39, 41, 45, 58, 66, or 93 kDa
Band(s) present IgM	41, 39, 23 kDa
Interpretive information IgM immunoblot	For this assay, a positive result is reported when any 2 of or more of the following bands are present: 23, 39, or 41 kDa

**Table 2:** Ehrlichia Chaffeensis antibodies

Order Comment	Ordered by Lab
Ehrlichia Chaffeensis Ab,IgG	1:256
Ehrlichia Chaffeensis IgG Ref Com	Reference range: <1:64
Ehrlichia Chaffeensis Ab,IgM	<1:16
Ehrlichia Chaffeensis IgM Ref Com	Reference range <1:16
Comments	1:256 or greater …... Positive: Presence of IgG antibody to Ehrlichia Chaffeensis detected, suggestive of current or past infection

## Discussion

Lyme Carditis is a rare manifestation of Lyme disease with it occurring in approximately one out of every hundred Lyme disease cases reported [3]. It occurs in the early disseminated phase of the disease which occurs within weeks to months after infection. The bacteria Borrelia burgdorferi disseminates to the heart tissue with a myocardial biopsy showing transmural inflammatory infiltrates with lymphocytic infiltrates in the endocardium and occasionally spirochetes [4]. Most cases of Lyme carditis are seen to be benign with a resolution of all symptoms. However, when symptomatic it typically presents with fatigue, shortness of breath, lightheadedness, syncope, and palpitations [5]. Once diagnosed or clinical suspicion is high for Lyme carditis it is important to start early treatment as serious and fatal cardiac complications can develop such as myocarditis, cardiac tamponade, heart failure, cardiac arrhythmias, and sudden cardiac death [6]. The typical cardiac changes seen in EKG, which can occur are various degrees of atrioventricular blocks, bundle branch blocks, and ventricular and supraventricular arrhythmias.

Here, we report a case of a 70-year-old male with Lyme disease presenting with a second-degree, Mobitz type 1 AV block. Lyme disease often presents with early symptoms within a week such as erythema chronicum migrans in up to as many as 70%-80% of patients, and/or other flu-like symptoms such as fever, fatigue, malaise, lethargy, headache, myalgias, and arthralgias [7]. This patient did not have symptoms early in the course of this disease which had initially pushed the differential of Lyme disease down the list. Although originally denying any tick bites he later stated he may have had an encounter with a tick bite about two months ago while staying at his cabin. He usually takes precautions by wearing long sleeves and using bug spray; however, due to the ongoing COVID-19 pandemic, he became complacent with outdoor activities.

Early during the patient's stay, we had a wide differential; however, due to the incidence in the region, there was enough clinical suspicion to order a tick panel to determine if the cause of the patient's AV block may be due to Lyme disease. Current guidelines for Lyme disease testing are primarily based on its textbook presentation and symptomatology, with geography playing a secondary role. When the initial presenting feature is non-specific such as in our patient with AV block, it may be worthwhile for practitioners to use incidence data of the patient’s geographical location, or recent travel, to help guide the appropriateness of testing. The CDC considers a state to be high incidence when it has at least 10 confirmed cases per 100,000 on average for three reporting years. Using this criterion, 16 states from 2018 to 2020 were considered high incidence. Our specific case took place in New York, which is labeled high incidence with an average of 17.3 cases per 100,000 (from 2018 to 2020) [8]. The tick panel at our institution tests not only for Lyme disease but also other tick-borne zoonoses such as Babesia microti, Ehrlichia chaffeensis, and Anaplasma phagocytophilum. The patient's Lyme total antibody screen results were positive and following the positive two-tiered testing approach per the Infectious Diseases Society of America guidelines, a Lyme western blot was ordered which resulted in IgG and IgM being detected [9]. The patient's Anaplasma and Babesia antibody tests came back negative however the Ehrlichia IgG Ab was positive with a value of 1:256. This suggested the patient was coinfected by two different Zoonotic infections.

## Conclusions

Lyme disease can have various atypical presentations with most cases being self-limiting but some being more malignant and dangerous if undiagnosed as in this patient. In highly endemic areas such as the northeast and Midwest United States, early recognition and treatment of Lyme disease is important for the prevention of long-term complications of disseminated infection.
